# Prediction of the minimum spacer thickness required for definitive radiotherapy with carbon ions and photons for pelvic tumors: an in silico planning study using virtual spacers

**DOI:** 10.1093/jrr/rrab047

**Published:** 2021-05-31

**Authors:** Masayoshi Yamada, Yuya Miyasaka, Takayuki Kanai, Hikaru Souda, Ken Uematsu, Rei Matsueda, Natsuko Yano, Shohei Kawashiro, Hiroko Akamatsu, Mayumi Harada, Yasuhito Hagiwara, Mayumi Ichikawa, Hiraku Sato, Kenji Nemoto

**Affiliations:** Department of Radiation Oncology, Yamagata University Faculty of Medicine, 2-2-2, Iida-Nishi, Yamagata 990-9585, Japan; Department of Heavy Particle Medical Science, Yamagata University Faculty of Medicine, 2-2-2, Iida-Nishi, Yamagata 990-9585, Japan; Department of Radiation Oncology, Yamagata University Faculty of Medicine, 2-2-2, Iida-Nishi, Yamagata 990-9585, Japan; Department of Heavy Particle Medical Science, Yamagata University Faculty of Medicine, 2-2-2, Iida-Nishi, Yamagata 990-9585, Japan; Department of Radiation Oncology, Yamagata University Faculty of Medicine, 2-2-2, Iida-Nishi, Yamagata 990-9585, Japan; Department of Radiation Oncology, Yamagata University Faculty of Medicine, 2-2-2, Iida-Nishi, Yamagata 990-9585, Japan; Department of Radiation Oncology, Yamagata University Faculty of Medicine, 2-2-2, Iida-Nishi, Yamagata 990-9585, Japan; Department of Radiation Oncology, Yamagata University Faculty of Medicine, 2-2-2, Iida-Nishi, Yamagata 990-9585, Japan; Department of Radiation Oncology, Yamagata University Faculty of Medicine, 2-2-2, Iida-Nishi, Yamagata 990-9585, Japan; Department of Radiation Oncology, Yamagata University Faculty of Medicine, 2-2-2, Iida-Nishi, Yamagata 990-9585, Japan; Department of Radiation Oncology, Yamagata University Faculty of Medicine, 2-2-2, Iida-Nishi, Yamagata 990-9585, Japan; Department of Radiation Oncology, Yamagata University Faculty of Medicine, 2-2-2, Iida-Nishi, Yamagata 990-9585, Japan; Department of Radiation Oncology, Yamagata University Faculty of Medicine, 2-2-2, Iida-Nishi, Yamagata 990-9585, Japan; Department of Radiation Oncology, Yamagata University Faculty of Medicine, 2-2-2, Iida-Nishi, Yamagata 990-9585, Japan

**Keywords:** spacer, in silico, planning study, carbon-ion radiotherapy (C-ion RT), intensity-modulated radiotherapy (IMRT)

## Abstract

We aimed to predict the minimum distance between a tumor and the gastrointestinal (GI) tract that can satisfy the dose constraint by creating simulation plans with carbon-ion (C-ion) radiotherapy (RT) and photon RT for each case assuming insertion of virtual spacers of various thicknesses. We enrolled 55 patients with a pelvic tumor adjacent to the GI tract. Virtual spacers were defined as the overlap volume between the GI tract and the volume expanded 7–17 mm from the gross tumor volume (GTV). Simulation plans (70 Gy in 35 fractions for at least 95% of the planning target volume [PTV]) were created with the lowest possible dose to the GI tract under conditions that meet the dose constraints of the PTV. We defined the minimum thickness of virtual spacers meeting D2 cc of the GI tract <50 Gy as ‘MTS’. Multiple regression was used with explanatory variables to develop a model to predict MTS. We discovered that MTSs were at most 9 mm and 13 mm for C-ion RT and photon RT plans, respectively. The volume of overlap between the GI tract and a virtual spacer of 14 mm in thickness (OV14)-PTV was found to be the most important explanatory variable in the MTS prediction equation for both C-ion and photon RT plans. Multiple *R*^2^ values for the regression model were 0.571 and 0.347 for C-ion RT and photon RT plans, respectively. In conclusion, regression equations were developed to predict MTS in C-ion RT and photon RT.

## INTRODUCTION

Definitive radiotherapy (RT) for abdominal and pelvic tumors is often difficult to prescribe for the entire tumor. The gastrointestinal (GI) tract, including the small and large intestines, is a radiosensitive organ and the tolerable dose for the GI tract is usually considered to be about 50 Gy [1]. On the other hand, several previous studies have shown the utility of particle therapy for various malignant tumors (e.g. chordoma, hepatocellular carcinoma and rectal carcinoma [post-operative pelvic recurrence]), and the prescribed dose for these tumors is generally greater than 50 Gy (relative biological effectiveness [RBE]) [2– 4]. Therefore, if a tumor is adjacent to the GI tract, the dose for the tumor must be reduced to a dose below the tolerable dose of the GI tract. The use of spacers enables separation of the tumor and the GI tract. Therefore, it is possible to safely achieve a definitive dose for the tumor by using spacers.

Several studies have demonstrated the effectiveness of Gore-tex® sheets, a tissue expander and omentum as spacers for abdominal and pelvic tumors [5– 10]. However, several complications (e.g. migration and obstruction) have also been reported [11,12]. Absorbable spacers that have been developed in recent years are designed to maintain their thickness for about two months and then be gradually absorbed, so that dose reduction to surrounding important organs can be maintained during the irradiation period [13,14]. In addition, it has been reported that serious acute and late adverse events do not occur [15], and absorbable spacers can be used under health insurance for particle therapy in Japan.

A previous work by our group demonstrated that there were differences in dose reduction effects of RT modalities on the GI tract using simulation plans created by pre-spacer computed tomography (CT) and post-spacer CT for carbon-ion (C-ion) RT, proton RT and photon RT in cases of abdominal and pelvic tumors adjacent to the GI tract with insertion of greater omentum spacers (non-absorbable spacers) [16]. However, no study has shown the appropriate distance between the tumor and GI tract for insertion of spacers and the thickness of spacers that would allow a definitive dose to be delivered to the tumor within the tolerable dose of the GI tract.

The aim of this study was to predict the minimum thickness of virtual spacers (MTS; i.e. the minimum distance between the tumor and the GI tract) for C-ion RT and photon RT that can satisfy the dose constraint described below by creating simulation plans with both C-ion RT and photon RT for each case assuming insertion of virtual spacers of various thicknesses. The results of this study will be useful for predicting whether spacers should be inserted prior to treatment planning and for determining the thickness of the spacers required prior to surgery in cases in which spacers should be inserted.

## MATERIALS AND METHODS

### Study population

In this study, in order to use the experimental approach described later, we considered that cases that were imaged by simple CT with a slice thickness of 1 mm were desirable, and we decided to use cases in which PET-CT was performed. We searched the image database for PET-CT at Yamagata University Hospital for images obtained between April 2011 and September 2020 and found 19 332 cases. ​Among those cases, 55 cases of primary bone and soft tissue tumors and post-operative recurrent tumors (bone and soft tissue tumors, GI tumors, gynecologic tumors and urinary tumors) with pelvic tumors less than 7 mm from the GI tract (i.e. cases with duplication of the planning target volume [PTV] with the GI tract) were selected in chronological order. This study was a hypothetical design and did not exclude cases based on tumor size or the presence of metastasis. This study was approved by the Institutional Review Board of our institution. PET-CT images were acquired using Biograph 64 (Siemens Medical Solutions, Erlangen, Germany). Imaging was performed with all patients in the supine position.

### Delineations and treatment plans

All delineations and treatment plans were created by one certified radiation oncologist with 6 years of radiation therapy experience and were confirmed by three medical physicists with more than 1 year of C-ion RT experience.

Target delineations were basically performed according to a previous work by our group [16]. MIM ver. 6.4.6 (MIM Software Inc., Cleveland, OH) was used for contouring. The gross tumor volume (GTV) was delineated as the macroscopic tumor on CT and/or 18F-fluorodeoxyglucose (FDG)-PET. The clinical target volume (CTV) was the same as the GTV. The PTV included the CTV with a 7-mm margin for possible positioning errors. When the PTV was adjacent to the skin, we allowed a volume of 5 mm from the body surface to be subtracted from the PTV. The GI tract (small bowel, colon and rectum) was defined as organs at risk (OARs). The GI tract was delineated in the range expanded 10 mm to the cranial and caudal sides from the PTV.

**Fig. 1.**
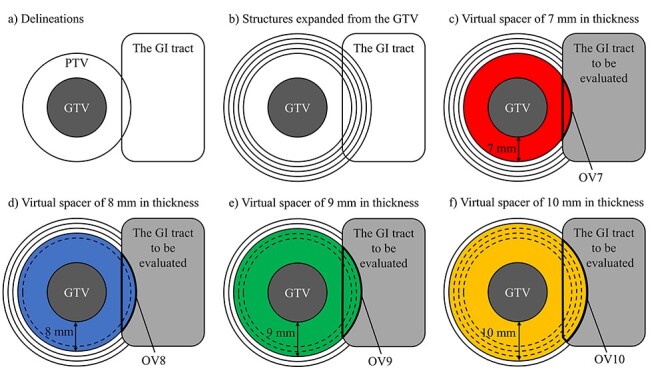
Schematic diagram of the delineations: (a) Delineations, (b) Structures expanded from the GTV, (c) Virtual spacer of 7 mm in thickness, (d) Virtual spacer of 8 mm in thickness, (e) Virtual spacer of 9 mm in thickness and (f) Virtual spacer of 10 mm in thickness. The structure with a red, blue, green and orange background shows the virtual spacer of 7 mm in thickness, that of 8 mm in thickness, that of 9 mm in thickness and that of 10 mm in thickness, respectively. The structure with a gray background shows the GI tract to be evaluated. Structures surrounded by a thick line show OVx (c) OV7, d) OV8, (e) OV9 and (f) OV10. ^*^OVx was defined as the volume of overlap between the GI tract and a virtual spacer of x mm in thickness.

In this study, virtual spacers of various thicknesses were created in order to determine the minimum distance between the tumor and the GI tract that enables a definitive dose to be delivered to the tumor within the tolerable dose of the GI tract. Fig. 1 show a schematic diagram of the delineations. Structures extended by 7–17 mm (at 1-mm intervals) in three dimensions from the GTV were created, and virtual spacers were defined as structures with the GTV subtracted. The GI tract to be evaluated was defined as the delineated GI tract with virtual spacers subtracted. OVx was defined as the volume of overlap between the GI tract and a virtual spacer of x mm in thickness.

C-ion RT plans were created on Raystation 9B (RaySearch Laboratories AB, Stockholm, Sweden) using a spot scanning technique as the irradiation method and a single-field uniform-dose (SFUD) optimization technique as the optimization method [17]. RBE-weighted dose distributions were calculated by using the modified microdosimetric kinetic model (modified MKM) [18]. The dose was calculated using the triple Gaussian-based pencil beam algorithm [19].

Photon RT plans were created on EclipseTM ver.15.1 (Varian Medical Systems, Palo Alto, CA). Photon RT plans were calculated using fixed-field intensity-modulated radiotherapy (IMRT) with 10 MV photons. The dose was calculated using an analytical anisotropic algorithm [20].

Although the prescription dose is usually reduced for definitive irradiation of pelvic tumors due to the adjacent GI tract, in this study, 70 Gy (RBE) in 35 fractions to the PTV was prescribed in all plans as a representative of the definitive dose based on a previous work by our group [16]. These plans were normalized to meet the prescribed dose for at least 95% of the PTV. The dose constraint for the PTV was the minimum dose to the ‘hottest’ 2% of the volume (D2%) < 77 Gy (RBE). All plans were created with the lowest possible dose to the GI tract under conditions that meet the dose constraint for the PTV. We also visually confirmed that there were no high dose areas outside the delineations described above. We aimed to quantify the thickness of virtual spacers that could meet the dose constraints of the minimum dose received by the most exposed 2 cc volume of the organ (D2 cc) of the GI tract <50 Gy (RBE). In this study, we defined the minimum thickness of the virtual spacers meeting the above conditions as 'Minimum Thickness of Spacers’ (MTS).

**Fig. 2.**
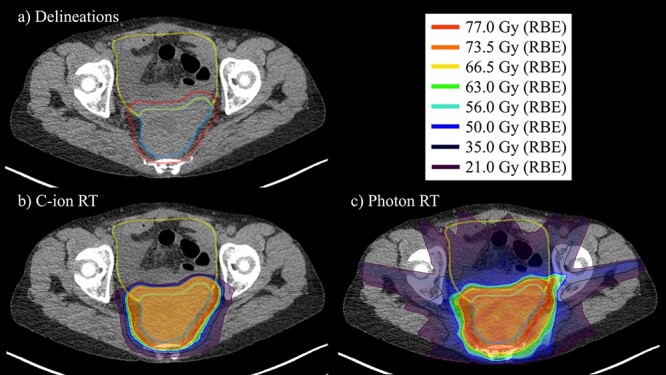
Examples of dose distributions: (a) delineations, (b) C-ion RT plans and (c) photon RT plans. The lines show GTV (blue), PTV (red) and GI tract (yellow).

For each plan, we chose a coplanar beam arrangement that minimizes the dose to the GI tract as much as possible. C-ion RT plans consisted of two to four beams, and photon plans consisted of four to seven beams. The optimal number of beams and beam angles were investigated so that D2 cc of the GI tract is minimized in each plan. All of the plans for C-ion RT and photon RT were calculated with the assumption of using a rotating gantry. We thought that replacing the electron density of the virtual spacers in the pelvic cavity with a value corresponding to absorbable spacers would make the MTS values more reasonable. Akasaka *et al.* reported that absorbable spacers are equivalent to water [21]. Therefore, we decided to replace the virtual spacer in the pelvic cavity with a relative electron density of 1.0167 g/cm^3^, which is equivalent to water, for the treatment plan. First, a treatment plan was made by replacing the virtual spacer of 7 mm in thickness with the above electron density, and then another treatment plan was made by increasing the thickness of the virtual spacer by 1 mm until the dose constraint was satisfied. Fig. 2 show examples of the dose distributions on C-ion RT and photon RT plans.

### Dose evaluation

Dose volume histograms (DVHs) were generated for the PTV and all OARs. D98% (minimum dose to the ‘hottest’ 98% of the volume), Dmean (mean dose), D50% (median dose), D2% and homogeneity index (HI) of the PTV were evaluated in all plans for C-ion RT and photon RT. HI was calculated by the following equation: HI = (D2%–D98%)/D50%. Smaller values for HI correspond to a more homogeneous dose across the target volume, and values close to 0 are optimal.

MIM was used for dose evaluation. We tested the differences in MTSs and relationships between the patients' parameters and MTSs for C-ion RT and photon RT.

### Statistical analysis

The differences in MTSs and HIs for C-ion RT and photon RT were analyzed statistically by Wilcoxon’s signed-rank test, and the relationships between patient parameters and MTS were statistically analyzed by single and multiple linear regression analyses. Correlations were determined by a statistic called the correlation coefficient (*R*), which represents the strength of the putative linear association between the variables in question. The multiple coefficients of determination (*R*^2^) value indicates the probability of fitting the regression equation for explaining the relationships between MTS and the included explanatory variables on a scale of 0–1. Multiple regression was used to develop a statistical model for the prediction of MTS. The linear relationships between each predictor variable and the response variable, MTS, were also assessed with a scatter plot matrix. The linear model to be fitted was as follows:

(1) \begin{equation*} {\rm{MTS}}=\beta_{0}+\beta_{1}{\rm{x}}_{1}+\beta_{2}{\rm{x}}_{2}+\beta_{\rm{i}}{\rm{x}}_{\rm{i}}+...+\beta_{\rm{n}}{\rm{x}}_{\rm{n}}+\epsilon \end{equation*}

In equation (1), β_0_ represents the intercept term, β_i_ represents the parameter estimates for the explanatory variables x_i_ included in the model, and ε is the error associated with the model. The stepwise method sequentially adds and removes explanatory variables to the model on the basis of their contributions to the overall fit [22]. In the multiple regression equation, the sign of the standard partial regression coefficient represents the direction of change in MTS and its absolute value represents the strength of the impact on MTS.

In this study, two patients' parameters, PTV and OV14-PTV, were used as explanatory variables for MTS in single and multiple linear regression analyses. This is because PTV indicates the volume of the target and OV14-PTV indicates the volume of the GI tract surrounding the PTV. OV14-PTV, the GI tract with a thickness of ‘7 mm’ in contact with the PTV, was used as an explanatory variable for MTS because the PTV margin is ‘7 mm’.

We calculated that 55 patients would need to be observed in order for the study to create a regression equation for MTS with 80% power and at a two-sided significance level of 0.05 with the use of multiple linear regression analysis. All statistical tests were performed using the R statistical package v.4.0.2, with a significance level set at p < 0.05.

## RESULTS

Clinical characteristics of the patients are shown in Table 1. Status of tumors was primary lesion, lymph node recurrence, local recurrence and peritoneal dissemination in 6, 12, 20 and 17 cases, respectively. Classification of tumors was GI tumors, gynecological tumors, urological tumors and osteochondral tumors in 20, 21, 4 and 10 cases, respectively.

**Table 1:** Clinical characteristics of the patients

Characteristic	Number of patients
**Status and localization of tumors**	
Primary lesion	6
Sacrum	5
Presacral space	1
Lymph node recurrence	12
Right or left iliac region	8
Pararectal region	3
Presacral region	1
Local recurrence	20
Right or left side of the pelvic region	8
Pelvic floor	7
Presacral space	5
Peritoneal dissemination	17
Right or left side of the pelvic region	9
Pelvic floor	7
Presacral space	1
**Classification of tumors**	
Gastrointestinal tumors	20
Rectal cancer	10
Ascending colon cancer	3
Sigmoid colon cancer	2
Appendix cancer	1
Rectal carcinoid	1
Rectal gastrointestinal stromal tumor	1
Rectal neuroendocrine tumor	1
Small intestinal cancer	1
Gynecological tumors	21
Ovarian cancer	10
Cervical cancer	6
Endometrial cancer	3
Peritoneal cancer	1
Uterine sarcoma	1
Urological tumors	4
Ureteral cancer	3
Bladder cancer	1
Bone and soft tissue tumors	10
Liposarcoma	3
Undetermined pathology	3
Chordoma	2
Neurogenic tumor	1
Rhabdomyosarcoma	1

Volumes and dose parameters in C-ion RT and photon RT plans are shown in Table 2. Volumes of the PTV, OV7, OV14 and OV14-PTV varied. The dose constraint for the PTV (D2% < 77 Gy [RBE]) could be met in all treatment plans that were normalized to meet the prescribed dose for at least 95% of the PTV. HI was significantly lower in C-ion RT plans than in photon plans (one-sided p-value <0.001). This means that a more homogeneous dose across the target volume could be delivered by C-ion RT than by photon RT.

**Table 2:** Volumes and dose parameters in C-ion RT and photon RT plans. Values are means ± SD with ranges in parenthesis

	C-ion RT plans	Photon RT plans
Volumes (cc)		
PTV	91.01 ± 141.93 (9.77–874.96)	
OV7	13.19 ± 18.15 (0.04–88.43)	
OV14	37.63 ± 39.24 (2.19–191.39)	
OV14-PTV	24.44 ± 21.69 (2.15–102.97)	
Dose parameters of PTV		
D98% (Gy)	67.20 ± 1.73 (61.40–69.85)	67.65 ± 0.79 (65.88–68.88)
Dmean (Gy)	71.74 ± 1.16 (70.40–74.18)	73.61 ± 0.61 (72.03–74.72)
D50% (Gy)	71.92 ± 1.31 (70.39–74.61)	74.03 ± 0.79 (72.26–75.40)
D2% (Gy)	73.07 ± 1.73 (70.76–76.40)	75.93 ± 0.64 (73.27–76.92)
HI	0.08 ± 0.04 (0.01–0.19)	0.11 ± 0.02 (0.06–0.14)

Notes: C-ion: carbon-ion; D_2%:_ minimum dose to ‘hottest’ 2% of the volume; D50%: median dose; D_98%:_ minimum dose to ‘hottest’ 98% of the volume; Dmax: maximum dose; D_mean:_ mean dose; HI: homogeneity index (HI = (D2%-D98%) / D50%); OVx: volume of overlap between the GI tract and a virtual spacer of x mm in thickness; RT: radiotherapy; SD: standard deviation.

**Fig. 3.**
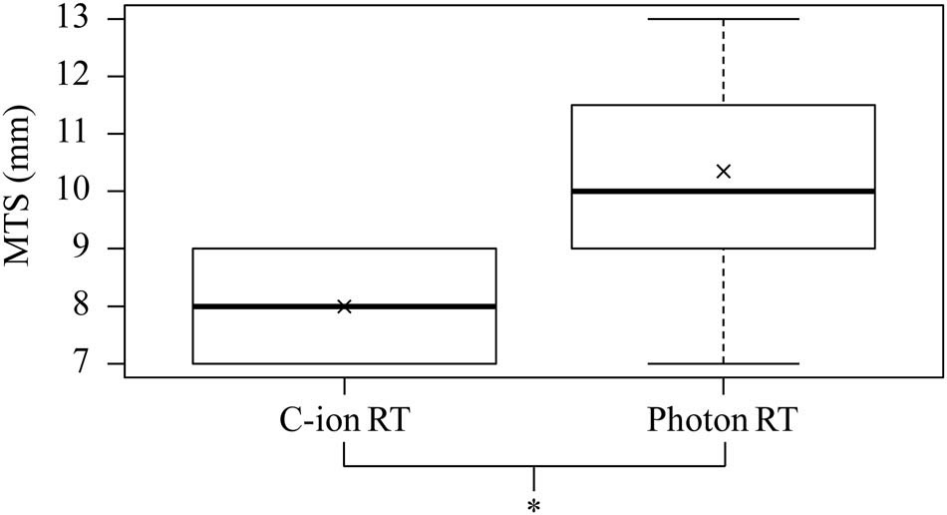
Multiple box plots of MTS (mm) in C-ion RT and photon RT plans. The box plots represent data with boxes ranging from the 25th to 75th percentiles of the observed distribution of values. Horizontal lines represent median values for MTS. X marks represent mean values for MTS. Whiskers span minimum to maximum observed values with algorithm-defined outliers. ^*^one-sided p-value <0.001(Wilcoxon’s signed-rank test).

Multiple box plots of MTS in C-ion RT and photon RT plans are shown in Fig. 3. In C-ion RT plans, the numbers of patients with MTSs of 7, 8 and 9 mm were 18, 19 and 18, respectively. In photon RT plans, the numbers of patients with MTSs of 7, 8, 9, 10, 11, 12 and 13 mm were 3, 4, 11, 11, 12, 7 and 7, respectively. MTS was significantly smaller in C-ion RT plans than in photon plans (one-sided p-value <0.001).

**Fig. 4.**
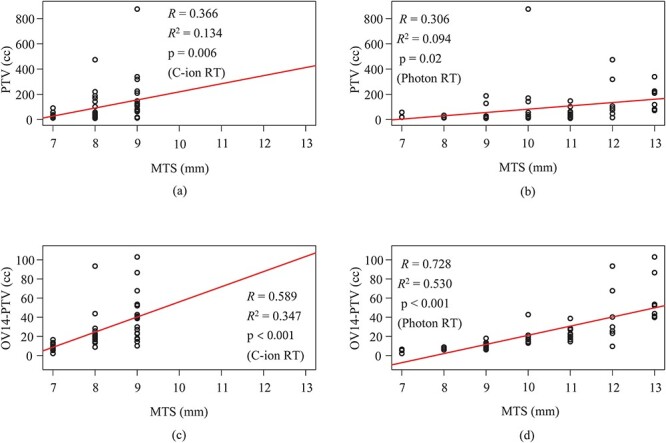
Correlations between MTS and patients’ parameters (PTV and OV-PTV) for C-ion RT and photon RT plans. Scatterplots of MTS and PTV for (a) C-ion RT and (b) photon RT plans and scatterplots of MTS and OV14-PTV for (c) C-ion RT and (d) photon RT plans are shown. The correlation coefficient (*R*), coefficient of determination (*R*^2^) and p-value are shown in (a)–(d), respectively.

With the use of single linear regression analysis, we found correlations between MTS and patients' parameters (PTV and OV14-PTV) for C-ion RT and photon RT plans, as shown in Fig. 4. *R* values between MTS and PTV were 0.366 for C-ion RT plans and 0.306 for photon RT plans. *R* values between MTS and OV14-PTV were 0.589 for C-ion RT plans and 0.728 for photon RT plans. Positive correlations of PTV and OV14-PV with MTS were obtained for C-ion RT and photon RT plans. *R*^2^ values between MTS and PTV were 0.134 for C-ion RT plans and 0.094 for photon RT plans. *R*^2^ values between MTS and OV14-PTV were 0.347 for C-ion RT plans and 0.530 for photon RT plans.

In this study, a multiple linear regression analysis was performed to estimate patients’ parameters associated with MTS for C-ion RT and photon RT plans. The coefficient estimates and associated p-values for the predictor variable chosen by the stepwise regression method are shown in Table 3. For C-ion RT plans, the stepwise regression method produced the following model:

(2) \begin{equation*} {\rm{MTS}}=\beta_{0}+\beta_{1} \left({\rm{OV14-PTV}} \right) +\epsilon \end{equation*}

In Equation 2, ε represents the error associated with the model. The multiple *R^2^* value for this model was 0.347. For photon RT plans, the stepwise regression method produced the following model:

(3) \begin{equation*} {\rm{MTS}}=\beta_{0}+\beta_{1} \left({\rm{OV14-PTV}}\right) +\beta_{2}\left({\rm{PTV}}\right) +\epsilon \end{equation*}

The multiple *R^2^* value for this model was 0.571.

OV14-PTV was associated with an increase in MTS for both C-ion RT and photon RT plans (both p-values <0.001). PTV was associated with a decrease in MTS for photon RT plans (p = 0.030). For C-ion RT plans, PTV was excluded from explanatory variables of MTS by the stepwise method. The absolute value of the standardized partial regression coefficient was the highest in OV14-PTV for both C-ion RT and photon RT plans. In other words, OV14-PTV was found to be the most important explanatory variable in the MTS prediction equation for both C-ion RT and photon RT plans.

## DISCUSSION

In a previous work by our group, we investigated the differences in dose reduction effects on the GI tract of greater omentum spacers in C-ion RT, proton RT and photon RT [16]. However, we were not able to determine the MTS that would allow a definitive dose to be delivered to the tumor within the tolerable dose of the GI tract. In this study, we used an experimental approach in which the volume of the GI tract is defined as the volume of the original GI tract minus the volume of the GTV extension, assuming that a virtual spacer is inserted. We used an experimental approach to define the volume of the GI tract to be evaluated as the volume of the original GI tract minus the volume expanded from the PTV, assuming the insertion of a virtual spacer. MTS should ideally be considered by developing treatment plans for multiple cases with tumors adjacent to the GI tract and determining whether a definitive dose can be delivered. However, because the volume and shape of the GI tract in contact with the tumor varies from case to case, we used the experimental approach described above. There has been no previous study in which such an approach was used, but this approach allows us to take into account different patient parameters (i.e. tumor volume and volume of the GI tract in contact with the tumor) in each case.

In this study, MTSs required to deliver the prescribed dose (70 Gy [RBE]) for at least 95% for the PTV and to meet D2cc of the GI tract <50 Gy (RBE) were at most 9 mm and 13 mm for C-ion RT and photon RT plans, respectively. Since the PTV was created from the GTV with an isotropic margin of 7 mm, the distances from the PTV to the GI tract to be evaluated were required to be at most 2 mm and 6 mm to reduce the dose from 70 Gy (RBE) to 50 Gy (RBE) in C-ion RT and photon RT plans, respectively. To the best of our knowledge, this study appears to be the first to show the distance between the tumor and GI tract at which a definitive dose can be delivered to the tumor.

With the use of single linear regression analysis, positive correlations of PTV and OV14-PTV with MTS were obtained for C-ion RT and photon RT plans. In addition, a regression equation was developed to predict MTS using the stepwise method in both C-ion RT and photon RT plans. Multiple regression analysis showed that MTS could be predicted from OV14-PTV in C-ion RT plans and from OV14-PTV and PTV in photon RT plans. OV14-PTV was also found to be most strongly associated with an increase in MTS in both C-ion RT and photon RT plans.

In cases of definitive RT for a tumor adjacent to the GI tract, the decision for performing surgical spacer placement is usually based on the actual planned dose distribution or the experience of the radiation oncologist. In clinical use, the results of this study can help to predict whether spacers should be inserted prior to treatment planning and to determine the thickness of the spacers required prior to surgery in cases in which spacers should be inserted. In order to apply the results of this study to clinical practice, we considered the following flow. The GI tract and PTV are created and the PTV and OV14-PTV are identified. ​The predicted distance between the PTV and GI tract required to satisfy the dose prescription is the MTS predicted value derived by substituting PTV and OV14-PTV into the regression equation minus 7. However, since the multiple *R*^2^ values (i.e. the probability of fitting to the regression equation) of the regression developed in this study were not so high, we consider that the maximum value of 2/6 mm (C-ion RT/photon RT) of the distance between the PTV and GI tract to be evaluated should be used. In other words, the index of the distance between the PTV and the GI tract required to satisfy the dose prescription is to use the higher of the predicted MTS minus 7 and 2/6 mm (C-ion RT/photon RT). It is anticipated that spacer insertion is unnecessary ​if the distance between the PTV and GI tract is greater than the above index, and it is desirable to insert a spacer with a thickness equal to or greater than the above index when inserting a spacer.

In this study, static-IMRT (e.g. fixed-field IMRT) was used. This is because static-IMRT is considered to have a smaller value of mean dose to normal organs, a narrower region of low dose in normal organs and a non-inferior value of conformity index than helical-IMRT (e.g. volumetric intensity-modulated arc therapy [VMAT) [23– 25].

In this study, the distance to expand the virtual spacer was increased by 1 mm. Since 1 mm thick slices were used in CT or PET-CT images, we did not consider it reasonable to evaluate the GI tract by increasing the distance by less than 1 mm to create virtual spacers.

There are some limitations of this study. First, in cases of a tumor adjacent to the bladder, there may be cases in which the internal margin should be considered. In this study, we adopted an isotropic margin for easier comparison of treatment plans. Second, in three cases, a volume of 5 mm from the body surface was subtracted from the PTV in order to meet the tolerable dose of the skin based on the prescribed dose for at least 95% of the PTV. The decrease in the PTV on the skin side did not change the PTV adjacent to the GI tract. Therefore, these cases are not considered to have had any impact on the consideration in this study. Third, deformation of the GI tract caused by spacer insertion was not taken into account in this study. A deformed GI tract may be more widely adjacent to the PTV than the GI tract to be evaluated in this study. In that case, a thicker spacer may be required than the MTS values calculated from the regression equation in this study. Fourth, it is unclear what effect MTS has in cases of upper tumors adjacent to OARs (e.g. liver, kidney and spinal cord), which were not considered in this study. Fifth, since this study was conducted using conventional fractionated irradiation, the results of this study cannot be directly applied to hypo-fractionated irradiation, which is commonly used in C-ion RT.

**Table 3:** Multiple linear regression analysis using the stepwise method of patients’ parameters associated with MTS for C-ion RT and photon RT plans

	Multiple R^2^	Variable	Coefficient estimate (95%CI)	Coefficient p-value	Standardized partial regression coefficient
C-ion RT plans	0.347	Intercept	β_0_ = 7.463 (7.192–7.734)	< 0.001	0.000
		OV14-PTV	β_1_ = 0.022 (0.014–0.030)	< 0.001	0.589
Photon RT plans	0.571	Intercept	β_0_ = 8.957 (8.502–9.412)	< 0.001	0.000
		OV14-PTV	β_1_ = 0.068 (0.050–0.086)	< 0.001	0.895
		PTV	β_2_ = −0.003 (−0.003 – –0.006)	0.010	−0.263

Notes: CI: confidence interval; C-ion: carbon-ion; MTS: minimum thickness of spacers; OVx: volume of overlap between the gastrointestinal tract and a virtual spacer of x mm in thickness; PTV: planning target volume; RT: radiotherapy.

## CONCLUSION

In this study, multiple linear regression with MTS and patients’ parameters identified by the treatment plans resulted in the development of a predictive model of MTS in individual patients in C-ion RT and photon RT plans. In clinical use, we believe that the results of this study will be useful for predicting whether spacers should be inserted prior to treatment planning and for determining the thickness of the spacers required prior to surgery in cases in which spacers should be inserted.

## CONFLICT OF INTEREST

The authors declare they have no conflicts of interest.

## References

[1] Emami B, Lyman J, Brown A et al. Tolerance of normal tissue to therapeutic irradiation. Int J Radiat Oncol Biol Phys 1991;21:109–22. 2032882 10.1016/0360-3016(91)90171-y

[2] Tsujii H, Kamada T. A review of update clinical results of carbon ion radiotherapy. Jpn J Clin Oncol 2012;42:670–85. 22798685 10.1093/jjco/hys104 PMC3405871

[3] Kamada T, Tsujii H, Blakely EA et al. Carbon ion radiotherapy in Japan: an assessment of 20 years of clinical experience. Lancet Oncol 2015;16:e93–100. 25638685 10.1016/S1470-2045(14)70412-7

[4] Igaki H, Mizumoto M, Okumura T et al. A systematic review of publications on charged particle therapy for hepatocellular carcinoma. Int J Clin Oncol 2018;23:423–33. 28871342 10.1007/s10147-017-1190-2

[5] Fukumoto T, Komatsu S, Hori Y et al. Particle beam radiotherapy with a surgical spacer placement for advanced abdominal leiomyosarcoma results in a significant clinical benefit: particle beam with a surgical spacer. J Surg Oncol 2010;101:97–9. 19798696 10.1002/jso.21417

[6] Hoffman JP, Sigurdson ER, Eisenberg BL. Use of saline-filled tissue expanders to protect the small bowel from radiation. Oncology (Williston Park) 1998;12:51–4 discussion 54, 60, 62, passim. 9474587

[7] Komatsu S, Hori Y, Fukumoto T et al. Surgical spacer placement and proton radiotherapy for unresectable hepatocellular carcinoma. World J Gastroenterol 2010;16:1800–3. 20380016 10.3748/wjg.v16.i14.1800 PMC2852832

[8] Ogino T . Surgical organ displacement: what is the best “materials and methods” for proton radiotherapy? Chin J Cancer Res 2013;25:267–8. 23825900 10.3978/j.issn.1000-9604.2013.04.03 PMC3696705

[9] White JS, Biberdorf D, DiFrancesco LM et al. Use of tissue expanders and pre-operative external beam radiotherapy in the treatment of retroperitoneal sarcoma. Ann Surg Oncol 2007;14:583–90. 17094026 10.1245/s10434-006-9139-0

[10] Jesseph JM, Fitzek MM, Shahnazi K et al. Surgical organ displacement for proton radiotherapy. Transl Cancer Res 2012;1:247–54.

[11] Ogino T, Sekimoto M, Nishimura J et al. Intraluminal migration of a spacer with small bowel obstruction: a case report of rare complication. World J Surg Oncol 2012;10:30. 22309780 10.1186/1477-7819-10-30 PMC3293069

[12] Steinhagen E, Khaitov S, Steinhagen RM. Intraluminal migration of mesh following incisional hernia repair. Hernia J Hernias Abdom Wall Surg 2010;14:659–62. 10.1007/s10029-010-0708-6 20658162

[13] Sasaki R, Demizu Y, Yamashita T et al. First-In-Human Phase 1 Study of a Nonwoven Fabric Bioabsorbable Spacer for Particle Therapy: Space-Making Particle Therapy (SMPT). Adv Radiat Oncol 2019;4:729–37. 31673666 10.1016/j.adro.2019.05.002 PMC6817542

[14] Kimura M, Asai K, Iwata H et al. Impact on dose distribution and volume changes of a bioabsorbable polyglycolic acid spacer during chemo-proton therapy for a pediatric Ewing sarcoma. J Radiat Res (Tokyo) 2020;61:952–8. 32960269 10.1093/jrr/rraa087 PMC7674708

[15] Komatsu S, Demizu Y, Sulaiman NS et al. Space-making particle therapy for sarcomas derived from the abdominopelvic region. Radiother Oncol J Eur Soc Ther Radiol Oncol 2020;146:194–9. 10.1016/j.radonc.2020.02.021 32220700

[16] Yamada M, Sato H, Ieko Y et al. In silico comparison of the dosimetric impacts of a greater omentum spacer for abdominal and pelvic tumors in carbon-ion, proton and photon radiotherapy. Radiat Oncol 2019;14:207. 31752932 10.1186/s13014-019-1411-0 PMC6868713

[17] Lomax A . SFUD, IMPT, and plan robustness. Particle Radiotherapy Springer 2016;169–94.

[18] Inaniwa T, Furukawa T, Kase Y et al. Treatment planning for a scanned carbon beam with a modified microdosimetric kinetic model. Phys Med Biol 2010;55:6721–37. 21030747 10.1088/0031-9155/55/22/008

[19] Inaniwa T, Kanematsu N, Hara Y et al. Erratum: Implementation of a triple Gaussian beam model with subdivision and redefinition against density heterogeneities in treatment planning for scanned carbon-ion radiotherapy (Phys. Med. Biol. 59 5361). Phys Med Biol 2014;59:6305–5. 10.1088/0031-9155/59/18/5361 25157579

[20] Fogliata A, Nicolini G, Vanetti E et al. Dosimetric validation of the anisotropic analytical algorithm for photon dose calculation: fundamental characterization in water. Phys Med Biol 2006;51:1421–38. 16510953 10.1088/0031-9155/51/6/004

[21] Akasaka H, Sasaki R, Miyawaki D et al. Preclinical evaluation of bioabsorbable polyglycolic acid spacer for particle therapy. Int J Radiat Oncol Biol Phys 2014;90:1177–85. 25539373 10.1016/j.ijrobp.2014.07.048

[22] Dowdy S, Wearden S, Chilko D. Statistics for Research. Hoboken, New Jersey: John Wiley & Sons, 2011.

[23] Hsieh CH, Liu CY, Shueng PW et al. Comparison of coplanar and noncoplanar intensity-modulated radiation therapy and helical tomotherapy for hepatocellular carcinoma. Radiat Oncol 2010;5:40. 20492727 10.1186/1748-717X-5-40 PMC2881007

[24] Song JH, Son SH, Kay CS et al. Reducing the probability of radiation-induced hepatic toxicity by changing the treatment modality from helical tomotherapy to fixed-beam intensity-modulated radiotherapy. Oncotarget 2015;6:33952–60. 26376679 10.18632/oncotarget.5581 PMC4741816

[25] Kuo YC, Chiu YM, Shih WP et al. Volumetric intensity-modulated Arc (RapidArc) therapy for primary hepatocellular carcinoma: comparison with intensity-modulated radiotherapy and 3-D conformal radiotherapy. Radiat Oncol 2011;6:76. 21693003 10.1186/1748-717X-6-76 PMC3138395

