# Corrigendum to: Prediction of the minimum spacer thickness required for definitive radiotherapy with carbon ions and photons for pelvic tumors: an in silico planning study using virtual spacers

**DOI:** 10.1093/jrr/rrab062

**Published:** 2021-06-23

**Authors:** Masayoshi Yamada, Yuya Miyasaka, Takayuki Kanai, Hikaru Souda, Ken Uematsu, Rei Matsueda, Natsuko Yano, Shohei Kawashiro, Hiroko Akamatsu, Mayumi Harada, Yasuhito Hagiwara, Mayumi Ichikawa, Hiraku Sato, Kenji Nemoto

**Affiliations:** 1 Department of Radiation Oncology, Yamagata University Faculty of Medicine, 2-2-2, Iida-Nishi, Yamagata 990-9585, Japan; 2 Department of Heavy Particle Medical Science, Yamagata University Faculty of Medicine, 2-2-2, Iida-Nishi, Yamagata 990-9585, Japan

When this paper first published online, the author name Rei Mastueda was incorrect. It should have been Rei Matsueda. This has now been corrected online.

